# Insight into Mechanobiology: How Stem Cells Feel Mechanical Forces and Orchestrate Biological Functions

**DOI:** 10.3390/ijms20215337

**Published:** 2019-10-26

**Authors:** Chiara Argentati, Francesco Morena, Ilaria Tortorella, Martina Bazzucchi, Serena Porcellati, Carla Emiliani, Sabata Martino

**Affiliations:** 1 Department of Chemistry, Biology and Biotechnologies, University of Perugia, Via del Giochetto, 06126 Perugia, Italy; chiara.argentati89@gmail.com (C.A.); francesco.morena@unipg.it (F.M.); tortorella.i@hotmail.it (I.T.); martina.bazzucchi89@gmail.com (M.B.); serena.porcellati@unipg.it (S.P.); carla.emiliani@unipg.it (C.E.); 2 CEMIN, Center of Excellence on Nanostructured Innovative Materials, Via del Giochetto, 06126 Perugia, Italy

**Keywords:** stem cells, mechanotransduction, mechanosensing, regenerative medicine, ex-vivo stem cell models, computational tools, stem cell-biomaterial interaction

## Abstract

The cross-talk between stem cells and their microenvironment has been shown to have a direct impact on stem cells’ decisions about proliferation, growth, migration, and differentiation. It is well known that stem cells, tissues, organs, and whole organisms change their internal architecture and composition in response to external physical stimuli, thanks to cells’ ability to sense mechanical signals and elicit selected biological functions. Likewise, stem cells play an active role in governing the composition and the architecture of their microenvironment. Is now being documented that, thanks to this dynamic relationship, stemness identity and stem cell functions are maintained. In this work, we review the current knowledge in mechanobiology on stem cells. We start with the description of theoretical basis of mechanobiology, continue with the effects of mechanical cues on stem cells, development, pathology, and regenerative medicine, and emphasize the contribution in the field of the development of ex-vivo mechanobiology modelling and computational tools, which allow for evaluating the role of forces on stem cell biology.

## 1. Introduction

The knowledge that mechanical forces regulate tissue development and remodelling dates back more than a century ago, when Julius Wolff observed that bones trabeculae coordinated with the principal stress lines that are caused by daily physical loading and hypothesised that bone tissue is capable of adapting its architecture to the mechanical environment [1]. More recently, many research groups have demonstrated the role of tissue mechanics and the effects of different type of forces in development, stem cell differentiation [2,3], and more generally in cells’ physiology and diseases [4,5,6]. In this review, we discuss the general concepts of mechanobiology and highlight the effects of mechanical cues on stem cells, development, pathology, and regenerative medicine.

## 2. Mechanobiology: How Mechanical Forces are Translated in Biochemical Signals

In this section we discuss the molecular basis of mechanobiology.

### 2.1. General Concepts

Mechanobiology, at the cellular level, specifies how cells exert, sense, decipher, and respond to physical forces. At the molecular level, mechanobiology specifies how mechano-molecular players are recruited and interconnected together to activate a specific biological function [2,3,7].

**Figure 1:**
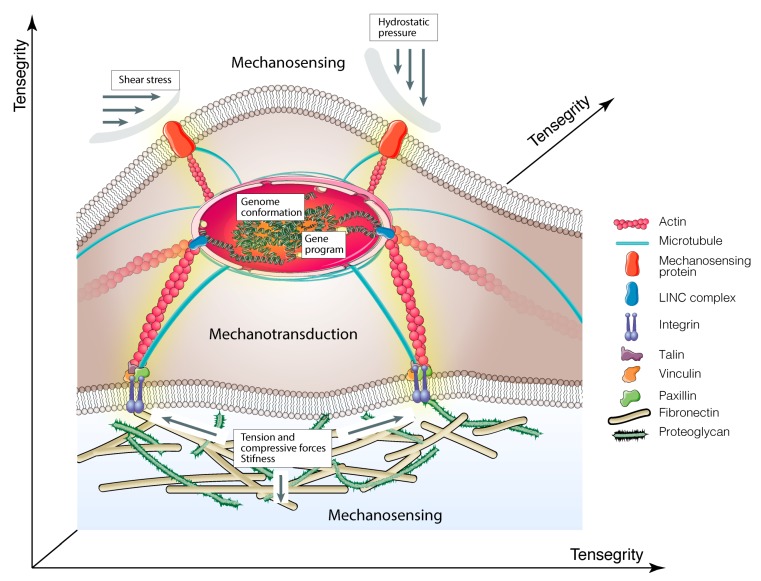
Schematic illustration of molecular basis of mechanobiology. Cartoon shows how mechanical cues are transmitted to the nucleus via integrins > focal adhesion complex > cytoskeletal components > nucleoskeleton. The yellow shadow indicates mechanotransduction signals.

These phenomena are the consequence of two main events, referred to as (i) *mechanosensing*, or the capacity of cells to sense physical cues and mechanical forces from the surrounding microenvironment and (ii) *mechanotransduction*, or the capacity of the cells to transduce either external forces into biochemical signals to elicit selected cell functions [2,3] or to intracellular molecular interaction into forces that influence the architecture and properties of the microenvironment [8] (see points 2.3 and 2.4, respectively) (Figure 1).

### 2.2. Tensegrity

Currently, the theoretical explanation of mechanobiology is based on the discovery that, in all cells, the cytoskeleton acts as a dynamic machine that collects the external forces applied to the cell from the microenvironment and responds by generating traction/compression forces transmitted to other molecular components inside or outside the cells. This model is based on the concept of “tensegrity” (tensional integrity), by which living cells organize their cytoskeleton as a hard-wired that immediately responds to external mechanical stresses stabilizing its form [9,10].

Tensegrity is a building principle, being originally described by the architect R.B. Fuller and pictured by the sculptor K. Snelson. While Fuller defined a tensegrity system “as structures that stabilize their shape by continuous tension or ‘tensional integrity’, rather than by continuous compression”, Snelson demonstrated that network structures may mechanically stabilize themselves through the use of tensile pre-stress forces [11,12,13]. In 1993, D. Ingber applied the term “tensegrity” to living organisms, suggesting a mechanical model where the cytoskeleton structure acts as a dynamic load-bearing pillar. This model, which is capable of recapitulating the events leading to cytoskeletal mechanics, cell shape, and movement, allowed for explaining how cells sense and respond to mechanical forces and, above all, how these two events are connected [14]. Hence, tensegrity predicts that cells respond straightaway to external mechanical stresses applied to the cells’ surface, through proteins that are physically connected to the cytoskeleton [14,15]. Additionally, in this mechanical model, molecules that are activated by changes in cytoskeletal architecture function in the “solid-state” and transduce mechanical stresses into biochemical signals and gene expression program within single living cells [15]. Therefore, all living organisms use “tensegrity” to mechanically stabilize their shape and integrate and balance their structure and function at all size scales, from the molecular level to organs [15,16]. This is a consequence of cytoskeleton tension that is transduced into an equilibrium of opposing forces that are dispersed through the network of cytoskeletal filaments. Generally, tension is generated within the actomyosin contractile microfilaments and is counteracted by microtubules, which are able to resist the compression forces [10,16,17,18]. The Ingberg model has later been confirmed and improved by many other researcher groups [8,15,19,20,21,22]. Among these, Cai and co-authors, starting from the observation that the cytoskeletal components are nonlinear cell mechanical supports, introduced the concept of “initial imperfections” in the original tensegrity model. This scheme provided a new intuitive method for understanding the load-bearing capacity and distribution of force into the cytoskeleton [23].

### 2.3. Mechanosensing

As mentioned above, all organisms have evolved structures, enabling them to recognize and respond to mechanical forces [4,24]. This cross-talk takes place at the macroscale level (e.g., in organs and tissues), at the microscale level (e.g., in single cells), and also at the nanoscale level (e.g., in molecular complexes or single proteins) [5]. At present, we know that the different types of forces orchestrate the control of all biological functions, including stem cells’ commitment, determination, development, and maintenance of cells and tissues homeostasis [4,24,25]. Table 1 summarizes the different mechanical properties and the proteins serving as transmitters in mediating these processes. 

### 2.4. Mechanotransduction

This section describes how cells sense the mechanical forces exerted by extracellular matrix (ECM) and neighbouring cells, and discusses how mechanical stimuli are transduced into biochemical signals to activate specific gene programmes and trigger cellular responses. The ability of cells to exert forces on the ECM or on other cells is also emphasized.

#### 2.4.1. Extracellular Matrix

The effects of ECM on cell functions have been extensively studied. Nowadays, it is well known that chemical, mechanical, and topographical cues of ECM control cell adhesion, shape, and migration, as well as the activation of signal transduction pathways orchestrating gene expression and dictating proliferation and stem cells’ fate [37,38]. The ECM is a structural macromolecular network that creates a scaffold for cells interactions and support [37,38,39]. It is composed of (i) solid components, consisting of fibrous proteins, (e.g., collagen, elastin, laminin, fibronectin), glycosaminoglycans (GAGs; e.g., hyaluronic acid), proteoglycans (PGs; e.g., chondroitin sulfate, heparan sulfate, keratan sulfate), and syndecans (see Table 2); (ii) soluble components, such as cytokines, growth factors, and several classes of proteases, like a metalloproteinases (see Table 2), all of which serve as mediators between ECM and cells [37,38,39,40,41,42]. Based on composition and structural organization, it is possible to distinguish two extensive ECM structures: the basement membranes, providing a two-dimensional support for the cells (mainly composed of laminin, collagen IV, nidogen and heparan sulphate) and the connective tissues that provide a fibrous three-dimensional scaffold to the cells that is mainly composed of fibrillar collagens, PGs, and GAGs [41,42,43,44]. 

The overall components confer topography, viscosity, and mechanical properties to ECM. In particular elastic fibers, fibrillar collagens, GAGs, and the related PGs provide the mechanical properties of ECM, while fibrous proteins provide tensile strength (collagens, elastin) [37,38]. Therefore, based on the composition, ECM has the characteristics of a “soft material”, easily deformable at low stresses, or of an “hard material”, which require greater stresses to generate deformation [2,37,38,39,40,41,42,43,44,45]. Interestingly, it seems that the resulting architecture provides a sort of ‘mechanical memory’, correlating with stem cells’ differentiation toward selected lineages [33,38,46,47].

According to its composition, ECM might also acquire a peculiar geometrical conformation providing topographic and mechanical stimuli, which are critical in modulating stem cells’ phenotype [37,38,44].

Notably, between ECM and stem cells exists a dynamic cross-talk, as stem cells may change the ECM composition and remodel the architecture either by the secretion of ECM structural components and matrix metalloproteinases, or by exerting mechanical forces through the cytoskeleton fibers. The challenge is to create a suitable cell microenvironment that generates mechanosensing/mechanotransduction signals and guide stem cells’ functions [8].

Based on the previous considerations, it is not surprising that alterations in specific ECM components or in regulatory players could have an impact on biochemical and physical properties of ECM, which leads to a disorganized network and, eventually, to organ dysfunctions. In particular, abnormal ECM composition has consequences on its mechanical properties and on the onset and progression of numerous diseases, such as cancer and fibrosis [45,48,49]. For instance, mutations in genes encoding for elastin or elastin-associated glycoproteins cause Williams and Marfan syndromes [50].

#### 2.4.2. Integrins

Among transmembrane proteins, the integrins family is the main class of proteins taking up ECM signals (see Table 2 for details) [51,52]. Moreover, their position, integrins serve as mediators of bidirectional signalling inside/outside cells [42]. The bond of integrins with ECM proteins (e.g., collagens, fibronectin, elastin, laminins) activate a specific association with intracellular proteins, such as those of the focal adhesion complex, which transduce mechanical cues from the ECM to the cell (and vice versa), thus modulating cells’ functions [52,53].

#### 2.4.3. Focal Adhesion

Focal adhesion (FA) complexes are composed of a family of proteins with several domains (e.g., calponin homology (CH), pleckstrin homology (PH), src homology 2 (SH2), src homology 3 (SH3), FERM and LIM domains [54,55,56]), binding directly or indirectly through actin-binding proteins to the cytoplasmic domains of integrins [57,58]. FA proteins are organized into nanoscale strata constituting a bridge between integrins and cytoskeleton [57,58].

Vinculin, paxillin, talin and focal adhesion kinase (FAK), are the major components of FAs (Table 2).

The recruitment of FA proteins to the FA complex is tightly dependent on the forces transmitted by ECM-integrins bonds [53]. For instance, cell stretching generated by the transmission of mechanical forces in response to ECM rigidity elicits vinculin-talin interaction [42,51,58,59,60,61]. Moreover, talin1 with the ROD domain is directly implicated in the initiation and stabilization of the cell-matrix adhesion process, as well as in the reinforcement of integrin–cytoskeleton connections in response to forces [57,62,63]. It is also likely that type and power of mechanical forces are able to influence the correct positioning and conformation of specific FA proteins [64]. Therefore, understanding how mechanical forces induce compositional changes in FAs might provide information regarding the molecular signals transduced and thus on the cells’ functions involved [64].

#### 2.4.4. Adherens Junctions

Mechanical forces propagate across tissues’ cells through cell-to-cell interactions that are orchestrated by specific protein complexes [65,66]. Among these, the complexes of Adherens Junctions (AJs) play a key role in several processes, such as tissue remodelling, morphogenetic developments, wound healing coordination, and tissue elongation [65,66]. The main adhesion proteins forming AJs belong to the cadherin family. Type I cadherins link intracellular AJs to actin filaments. They are the most ubiquitously expressed and include: E-cadherin, P-cadherin (epithelial cells), VE-cadherin (endothelial cells), and N-cadherin (all other non-epithelial cells), originating from the variable sequences in the extracellular domain of the protein [66] (Table 2). The conserved cadherin repeats domain contains calcium-binding sequences, essentials for switching “off”/“on” the adhesive function of cadherins. By this process, between two neighbouring cells, E-cadherin preferentially binds E-cadherin and N-cadherin to N-cadherin [65,67,68]. Catenins, nectins, and related proteins are other important players mediating cell-to-cell interactions [66,69,70] (Table 2).

#### 2.4.5. Cytoskeleton

The regulation of cytoskeletontension guarantees forces propagation within cells [16,117,118]. This is a dynamic structure composed of F-actin microfilaments (polymer of G-actin), microtubules (polymer of αβ−tubulin dimers; MTs), intermediate filaments (polymers of small cell type-specific peptides; IFs), and cross-linking proteins, providing a three-dimensional support for the cells and having a direct impact on all basic and specialized cells’ functions (Table 2 for details) [117]. The mechanical properties of the cytoskeleton are strictly related to the dynamic, geometry, and polarity of its components [117,119]. In particular, F-actin has a flexible structure, resists intracellular stresses, and, upon stretch, activates local tension and cell rigidity. On the other hand, MTs are rigid and, in general, highly susceptible to longitudinal-torsional vibration modes [117]. When microtubules are elongated, vibration modes increase the rotation, the translation, and the velocities of intracellular vesicles along the filaments [16]. Lastly, intermediate filaments are highly flexible and deformable [118].

Noteworthy, the cytoskeleton contractility is mainly guaranteed by a network of actomyosin fibers that originates from the interaction of F-actin and non-muscle myosin-II [119,120]. F-actin and myosin II form stress fibers of 10–30 nm, together with other actin-linking proteins (e.g., α-actinin, fascin, filamin, spectrin, dystrophin, Arp2/3, profilin, ADF/cofilin, fimbrin, profilin, villin, formin family, and tropomyosins) [119,120]. These stress fibers may associate the cell–ECM interface directly through FAs complex or indirectly through a network between stress fibers [119,120]. Tension and mechanical forces transmission to the nucleus is typically due to actomyosin fibers and it is regulated by the levels of phosphorylation of the myosin light chain [120,121].

#### 2.4.6. Nucleoskeleton

Mechanical cues arising either from the ECM and collected by integrins and FAs or from cell-to-cell contact and perceived by the AJs are both transmitted from the cytoskeleton fibers to the nucleus. They influence cytoplasmatic proteins causing their structural modification and shuttling them to the nucleus where they have a key role in orchestrating gene expression [122,123]. Among these proteins, there is a class of transcriptional factors that activate or repress mechanosensitive genes [124,125], as the case of YAP and TAZ [126,127] of Hippo pathway, which translocate to the nucleus in cells that are subjected to stiff stimuli [126,127]. The canonical function of YAP and TAZ is to transduce signals critical for driving stem cells’ fate and regeneration, whereas their altered activity is involved in aberrant cell mechanics transduction and in several diseases (e.g., atherosclerosis, fibrosis, pulmonary hypertension, inflammation, muscular dystrophy, and cancer) [128]. Other transcription factors, such as NKX-2.5, are activated in response to low tension and, once in the nucleus, they act as “mechanorepressor” of genes deputed to the maintenance of cells high-tension state [123].

Indeed, a direct nuclear-cytoskeletal link is critical in transmitting forces to the nucleus and eliciting biological responses to them. Many studies have pointed at the nucleoskeleton structure as the principal regulator of biochemical and physical connections between the nucleus and the cytoskeleton [125,129,130]. Within the nucleoskeleton, intermediate filament lamins A, B, and C, are the mechanical components of the inner nuclear membrane, which are directly associated with chromatin domains, thus regulating genome conformation and gene expression [131,132,133] (Table 2). The other component is the LINC complex [129]. This is a conserved molecular bridge that consists of different proteins spanning the nuclear membrane and connecting the lamins to the cytoskeleton [129,134]. In mammalian, the LINC complex is composed of proteins containing SUN domains (e.g., SUN1 and SUN2) and KASH domains (nesprin-1 and nesprin-2) [135,136] (Table 2). Mechanical signals propagating through the LINC complex induce conformational changes in nuclear proteins and they have a direct impact on chromatin structure and gene expression reprogramming [129,134].

## 3. Mechanobiology in Development and Pathology

The mechanosensing/mechanotransduction signalling cross-talk between stem cells and ECM is highlighted in development processes and in several diseases. Some examples are reported in the following.

### 3.1. Development

The developing of embryos consists in stem cells aggregation and organization into increasingly more complex structures and internal and external mechanical forces determine these events [137,138]. Furthermore, the correct positioning of stem cells during morphogenesis is guaranteed by the appropriate establishment of mechanical interactions among them and with their microenvironment ECM [137,138]. 

The first evidences of the role that mechanics play in developmental processes came from non-mammalians, such as Drosophila [5,139], avians [140], amphibians [141], and fish [142], in which became clear how eggs fertilization and maturation strongly depend on osmotic pressure gradients that influence cells shape [143,144]. On the other hand, significantly less is known about the influence of mechanical forces in the development of human embryos inside uterus because of the limited amount of material available for experimentations on animal models used for recapitulating human physiology [145] and because of ethical restrictions regarding human embryos manipulation [143]. Therefore, much of our knowledge on this topic relies on observations on close primate species [146,147,148,149] or on archival material [150]. Hence, taking advantage of some evolutionarily conserved mechanisms, human embryonic mechano-signalling have been recently characterized. These include i) key regulator genes [151]; ii) body axes establishment and local strains [152]; iii) geometrical influence on cell populations sorting [153,154]; and, iv) embryonic architecture and early signalling gradients [155]. Hydrostatic pressure (HP) is one of the mechanical forces involved in embryogenesis (both in early and late phases of development). At the blastocyst stage, the internal HP dictates the right definition of cell fate and embryonic size [156], while in later stages the pressure applied by the amniotic fluid appears to guide notochord extension by stimulating the underlying mesoderm [143,157].

At present, *in-vitro* models of embryogenesis seem to be the only tool for effectively understanding the processes regulating patterning, morphogenesis, and mechanobiology in the peri-implantation human embryo, as far as progresses in the possibility of working with human embryos are made [158,159,160,161]. Nevertheless, it will be necessary to wait more precise characterization of the embryos that they are expected to model, especially given that benchmarks based on mouse biology may not hold true in human, in order to understand if these models accurately recapitulate the molecular events happening in-vivo [143,162].

### 3.2. Pathology

Advances in mechanobiology suggest that alterations in cell mechanics, ECM structure, or mechanotransduction signals may contribute to the development of many diseases. As a matter of fact, aberrant mechanical signals, which are caused by changes in the physical and structural features of the cell microenvironment or by defects in how cells perceive mechanical inputs, have been associated with the pathogenesis of many diseases [128,163].

For example, clinical evidences show that alterations in cell−ECM interactions can cause cancer [164,165]. In many tumors, ECM production and stiffness are significantly increased when compared to healthy tissue [166,167,168,169]. It has been suggested that cancer stem cells increase ECM stiffness, encouraging metastatic activity, and that tumor stiffness hinders the activity of immune cells. Therefore, some clinical treatments use TGF-β inhibitors to reduce ECM proteins secretion and prevent further ECM changes [166]. In human cancers cells, YAP and TAZ have a supra-normal expression level as a cell response to mechanical inputs from the tumor microenvironment [127,128,170]. Likewise, the role of endogenous forces in regulating different neuronal functions is also well established [171,172,173]. Disruptions or alterations of cellular-mechanical properties are associated with neurological diseases, such as Alzheimer’s disease [174], spread axonal injury, spinal cord injury, concussion, and traumatic brain injuries [175]. It has been shown that the up-regulation of FA proteins, such as vinculin, talin, paxillin, and actin-crosslinking α-actinin, causes astrocytes activation and increases the expression of intermediates filaments, including Glial Fibrillary Acidic Protein, vimentin, and nestin [176]. Astrocytes’ hypertrophy and hyperplasia intensifies the stress on surrounding cells and the secretion of ECM proteins, such as collagen IV and laminin, which form a collagenous basement membrane scar, one of the major obstacles to axonal regeneration [177,178,179].

Alterations in mechanical signals are also key factors in the pathophysiology of cardiovascular diseases [180]. In particular, arterial stiffening is recognized as one of the key events in the progression of several cardiovascular diseases, including coronary heart disease, hypertension, atherosclerosis, and stroke [181,182]. Moreover, the high susceptibility of skin to mechanical forces, being exposed to different environmental insults as the most external body layer [183,184], has been correlated to many pathologies, including keloids, scleroderma, and psoriasis [184,185,186,187].

The role of mechanical forces is also well known in bone tissue as well as the effects of biophysical cues in osteoblast differentiation [188,189], mineralisation process, inhibition of osteoclast differentiation, and protection against osteolysis [190,191]. Actually, the malfunctioning of some of these processes appears to be implicated in osteoarthritis and osteoporosis [192]. Moreover, during osteoporosis, mechanotransduction appears to be compromised, as there is an altered distribution of integrin-based mechanosensory complexes regulating Cox-2 expression and PGE2 release in osteocytes [193].

Finally, the recent characterization of eyes mechanobiology has been fundamental in understanding their functioning, angiogenesis, pathologies progression, and therapeutic approaches efficacy [194,195,196]. For instance, ECM proteins that are secreted by the eye stroma in response to chronic inflammation might alter the mechanical integrity of the ECM, which leads to the activation of YAP/TAZ and β-catenin signalling pathways that, in turn, enhance the epidermal differentiation of the epithelium. This can lead to corneal squamous cell metaplasia, which causes blindness [197].

## 4. Mechanobiology on Stem Cells and Regenerative Medicine 

### 4.1. Mechanosensing/Mechanotransduction Signalling Drive Stem Cell Functions

The study of mechanobiology in stem cells is pivotal in understanding the molecular processes regulating stem cells’ homeostasis, self-renewal, pluri/multipotency, and also for its prospective applications in regenerative medicine [198,199]. To facilitate readers, Table 3 summarizes stem cells’ properties and hierarchy.

**Figure 2:**
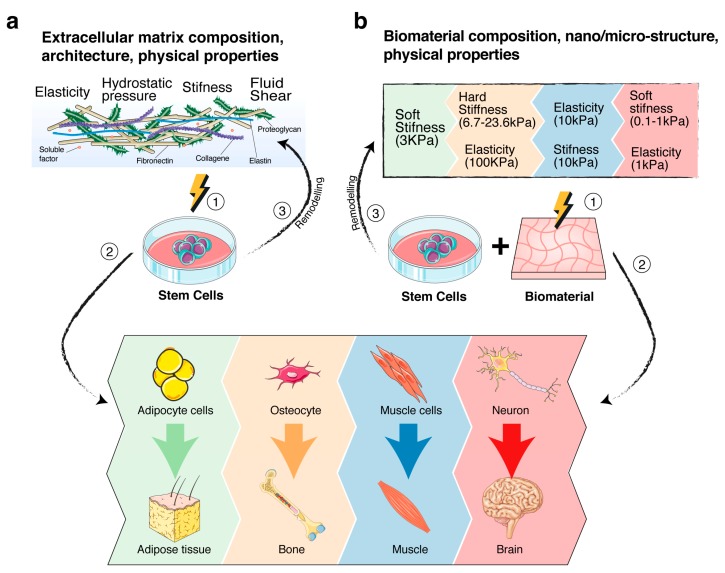
Schematic of the role of mechanosensing/mechanotransduction signalling on stem cell fate. (**a**) *Stem* cells-extracellular matrix *(ECM) cross-talk.* (1,2) Cartoon summarizes the different mechanical properties of ECM on driving the stem cell differentiation process toward a selected differentiation lineage, depending on the tailored composition, microstructure and physical cues of ECM. (3) Cartoon also shows the active role of stem cells on remodelling the ECM. The process, described in the text, is critical for the maintenance of stemness functions within the niche. (**b**) *Stem cells-biomaterials cross-talk*. (1,2) Cartoon summarizes some mechanical properties of biomaterials that have been directly involved in driving stem cell differentiation toward selected cell lineages. The colours correlate the mechanical property with the differentiation lineage [33,245,246,247,248]. (3) Schematic is also the active role of stem cells on remodelling the biomaterials. Modifications, induced by the cell secretion of ECM proteins and biomolecules, or/and by mechanical forces exerted by cells through the cytoskeletal fibers, have the challenge to recreate a stem cell microenvironment, suitable for stem cell functions.

Stem cell fate and behaviour are profoundly influenced by the microenvironment (also known as niche), in which they reside [60,223,224,225,226,227]. The niche is a tissue area consisting of ECM molecules, soluble proteins, such as cytokines and growth factors, and supporting cells [137,224,225,228,229,230,231], which, overall, generate peculiar chemical-physical cues having a critical role in maintaining stem cell self-renewal and pluri/multipotent properties [137,198,226,232]. Indeed, a mutual dynamic interaction exists between stem cells and their niche components. As matter of fact, stem cells influence their niche by secreting the above-mentioned bioactive molecules or by exerting mechanical forces through their cytoskeletal components (Figure 2a). Examples of this interplay clearly emerge by the plasticity of cancer stem cells in reorganizing their microenvironment [233,234], or by studies showing that the maintenance of pluripotency of naïve stem cells might be driven by the niche mechanotransducers signals that regulate the WNT/β-catenin pathway [235,236] or E-cadherin expression levels in stem cells [237,238]. Moreover, mechanical cues have been illustrated to elicit stem cells’ commitment and specification toward a selected stem cell differentiation lineage (Figure 2a). For example, it was demonstrated that ECM stiffness might directly control the lamin-A expression and thereby the specification lineages toward adipogenesis or osteogenesis of human mesenchymal stem cells (MSCs) [90]. Indeed, MSCs were the first adult stem cells used to demonstrate in-vitro the influence of matrix stiffness in adult stem cell differentiation, as they may be easily steered toward osteogenic differentiation or adipogenic differentiation, depending on the artificial support mimicking the ECM stiffer or softer characteristics, respectively [137,239,240]. On the other hand, the pioneering work by Engler and co-authors demonstrated that modulating the matrix elasticity might drive commitment to the lineage specification of MSCs [33]. The role of stiffness in controlling stem cells’ fate was also investigated by Aguilar and co-authors, which demonstrated that a 30- to 60-Pa stiffness of bone marrow was essential in the correct differentiation of megakaryocytes and for the generation of proplatelets [241]. Additionally, the delivery of a 10% static equibiaxial stretch to neural stem/progenitor cells was able to induce their differentiation toward oligodendrocytes, instead of astrocytes or neurons [242,243]. The mechanical characteristics of ECM and, in particular, the content of tropoelastin, seem to be also involved in the hematopoietic differentiation of hematopoietic stem cells [244].

### 4.2. Regenerative Medicine

From the aforementioned considerations, it is clear that the elucidation of mechanobiology processes in stem cells might have a direct impact on the development of innovative therapeutic tools for regenerative medicine application.

Regenerative medicine refers to the most innovative biotechnologies and therapies for the cure or replacement of defective/degenerate tissues and organs [211,249]. The paradigm of regenerative medicine is based on the potential of stem cells to maintain tissue homeostasis by replacing dead cells with newly differentiated progenies and releasing active molecules having a critical role in the regenerative processes (autocrine and paracrine actions) [200,211,250,251]. These biological properties are well-maintained, even when stem cells are transplanted into a host tissue/organ in-vivo (direct transplantation) [211,252,253], or when they are engineered with a therapeutic gene (gene-therapy) [254,255,256,257,258,259,260,261,262,263,264,265,266,267,268,269,270], or when they are combined with biomaterials to generate an ex-vivo tissue (tissue engineering) [240,271,272,273,274,275,276,277,278,279,280,281,282,283,284,285]. A further advance is the recent organ-on-a-chip technology [286], which recapitulate the human physiology through culturing stem cells in a tailored artificial tissue or in a single organ system (e.g., cardiac or lung tissues) [278,287,288,289].

### 4.3. Biomaterial System to Study the Effect on Stem Cell Fate by Mechanosensing/Mechanotransduction Signalling.

The use of biomaterials combined with stem cells for mimicking the ECM characteristics allow for establishing bio-hybrid substitutes for replacing defective tissue/organs in regenerative medicine therapeutic approaches [199,227] and allows investigating how mechanosensing and mechanotransduction signals may regulate stem cells homeostasis, self-renewal, and differentiation (Figure 2b).

The rationale of the use of biomaterials as stem cells support is based on the cross-talk taking place between them. Stem cells act on biomaterials releasing ECM proteins and bioactive molecules and exert forces through the cytoskeletal components to recreate their niche. Conversely, biomaterials act on stem cells through their intrinsic chemical-physical properties, which activate mechanosensing/mechanotransduction signalling and thereby modulate the stem cells fate (Figure 2b) [60,226,227,290,291,292].

Different types of natural (e.g., collagen, fibrin, silk) and synthetic (e.g., polylactic acid, polyesters, polyanhydrides, polyurethane) polymers have been manipulated to fabricate biocompatible films (two-dimensional) or scaffolds (three-dimensional) with tuneable properties to guide stem cells fate [281,283]. *In-vitro* synthetic models of stem cells niches have been developed to explore the effects of mechanical cues mode and magnitude in influencing stem cells’ proliferation and differentiation (as reviewed by Vining and Mooney) [137]. Modifications of shape and surface nanotopography of biomaterials have been demonstrated to modulate cell mechanotransduction axis and dictate selected stem cells’ functions [228,272,273,285,292]. In this regard, we demonstrated that Poly(L-lactide)acid (PLLA) polymer film and PLLA/Multi-Walled Carbon Nanotubes nanocomposite film activate two different mechanotransduction axes in human umbilical cord mesenchymal stem cells (hUCMSCs) [285]. In particular, hUCMSCs on PLLA were steered toward an epiblast (EPi)-like phenotype through the activation of NANOG and OCT3/4 by the mechanotransduction axis E-cadherin-F-actin/Myosin-IIA-Sun1. Alternatively, hUCMSCs on nanocomposite film were steered toward an endoderm (PrE)-like phenotype through the activation of GATA6 and GATA4 via N-cadherin−β-catenin linked to F-actin−Filamin-A−Lamin-A, as a second mechanotransduction axis [285].

Hydrogels with tailored elasticity and viscoelasticity characteristics have been produced to generate an *ex-vivo* model of organ/tissue for regenerative medicine applications [293,294]. Many laboratories have coated engineered extracellular matrix proteins on polyacrylamide hydrogels in order to modulate the stiffness characteristics to mimic in-vivo pathophysiological microenvironments for regenerative medicine [182,295,296].

Finally, advances in this field came from the extrusion-based bioprinting systems. This innovative technology combines stem cells and biomaterials to create bio-hybrid structures mimicking tissues or organs’ architecture and, therefore, allows more proficient basic research, development of pharmaceutics screening, and clinical translation [297].

### 4.4. Computational Tools to Study Stem Cell Mechanobiology

This review clarifies how mechanical properties of the ECM or of a biomaterial can influence stem cells fate, which is the final effect of step-by-step sequence having the first sign in the determination of the cells’ shape. The latter agrees with the biological basic concept that cells’ function and shape are strictly associated [298]. Therefore, the morphometric characterization of cells might represent a key analysis of cellular dynamics, as it takes the interactive reaction of cells with their microenvironment into account [299]. In this paragraph, we describe some current computational morphometric tools allowing for correlating shape and mechanical cues to cells’ functions. Bioinformatic offers the possibility to improve canonical cell shape and spread quantification [272,284,285], while taking into account the high variability that is given by the dynamic structures of the cells [299,300]. For example, due to a large number of filopodia (a signature of a highly dynamic cytoskeleton) in the cell, borders might present different and numerous irregularities. Moreover, contractile cells can withdraw from the focal adhesions at the margins, creating many membrane protrusions that increase the variability [299,300]. Cell spreading measures include geometric parameters for cells and nuclei, such as: area, perimeter, major and minor axis of fitted ellipse, and their ratio, convex hull parameters, radius and diameter of inscribed and circumscribed circles, and others [299,300]. Quantification is achieved by direct tracing and measuring of cell area and perimeter, shorter and longer cell axis, and other derivations, as reported in the Table equations (Table 4).

The table also shows common quantitative parameters that are used in morphometric analysis of cells cytoskeleton, all of which can be calculated using professional image processing software such as FIJI (an ImageJ-based package with a rich set of tools and plugin focused on scientific image analysis) ImageJ and CellProfiler (a Python based system) among free open-source software, IMARIS and MATLAB among closed source software [313,314,315,316,317].

**Figure 3:**
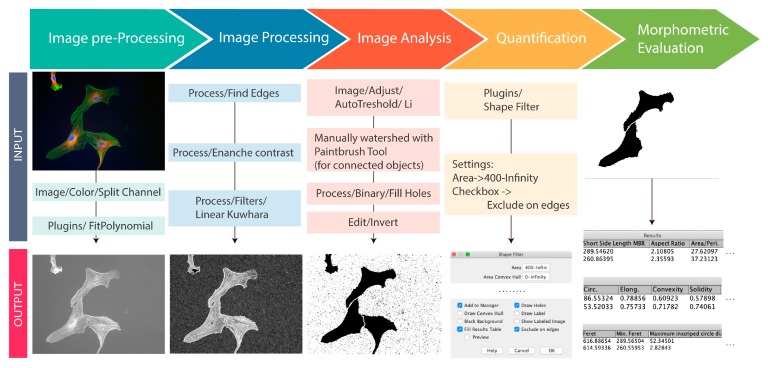
Schematic of a computational workflow for morphometric measurement of human adult mesenchymal stem cells. When working with RGB images, the first step is to split the colour channel into 8-bit grey colour. In most cases, it is necessary to correct the non-uniform illumination and then to convert the image into a binary image to make easier the identification of foreground objects. This allows analysis and computing statistics of the objects in the image. The procedure is summarized in the workflow. First, it is necessary to split the channels and to select the one of interest, then to fit polynomial plugins (https://imagejdocu.tudor.lu/doku.php?id=plugin:filter:fit_polynomial:start) to correct the illumination. Second, edges must be found with implemented function in FIJI to trace the outline of the objects and to enhance the contrast that allows gaining a higher contrast of the areas with lower local contrast. Before the image binarization, it is important to apply a Kuwahara filter, which is a noise-reduction filter that preserves edges, followed by auto threshold with Li method [324]. In some cases, it is necessary to watershed overlapping cells and this is achieved by different automated processes, as aforementioned; nevertheless, manually watershed remain the best and easiest choice when it is possible to clearly distinguish the contacts between objects. At times, because of the binary transformation imperfection recognized by the ideal threshold, certain background areas lie entirely in the foreground and they are referred as “holes” within the foreground objects. So, when the binary image is created, the area of the objects is filled and the final image inverted into an image with black objects on a white background. This allows the ‘Shape Filters’ plugin to analyse the objects of interest (cells or nuclei) and perform measurement, such as finding the area, perimeter, convex hull parameters etc. The original immunofluorescence image of human adult mesenchymal stem cells was from S. Martino laboratory.

The first step in the morphometric analysis of images is the identification and the selection of the object (i.e.,cell(s), nucleus(i)) within the image to calculate specific morphometric characteristics. In this regard, the segmentation of the studied object(s) and the reliable measurements of shape descriptors are two of the key processes for accurate image analysis [303]. Segmentation algorithms are often problematic when it comes to outline complex shapes and when there is overlapping cells [318]. Therefore, there are many different methods of segmentation to detect objects and boundaries: linear image filters, Laplacian-of-Gaussian or Gaussian filters, and mathematical segmentation methods [319,320,321]. Segmentation is required before calculating specific shape descriptors while using the outlines. After segmentation, biologically useful information must be extracted from the sample images. The best way to quantify cell morphology is using computer algorithms to mathematically quantify shape descriptors that represent specific cell morphology characteristics. ImageJ or FIJI may be used for such analyses [322,323]. For example, we report a simple FIJI (free open source) workflow for fluorescence image segmentation and morphometric measurement features with the help of the Shape filter plugin in FIJI (https://imagej.net/Shape_Filter) (Figure 3).

## 5. Conclusions

The elucidation of the mechanisms leading to crosstal-king between stem cells with their microenvironment uncovered networks of signals, which were activated by a single force or a synergy of forces, resulting in being critical in driving stem cells fate. Even if many studies are still ongoing, it is already clear that mechanical cues activated by the ECM and translated to the cell through mechanosensing/mechanotransduction signals represent a general scheme by which cells, tissues, organs, and whole organisms respond to external mechanical stimuli orchestrating their biological activity. Advances in the use of biomaterials as support for in vitro stem cells cultures to generate ex-vivo models of tissues and organs, together with computational systems, have highlighted the potentials of mechanobiology as a new therapeutic tool to be investigated for regenerative medicine applications.

## Figures and Tables

**Table 1:** Mechanical properties and biological mediators.

Mechanical Properties		Proteins	Ref.
Tension	Tensile forces refer to the external stimuli that tend to stretch cells, acting in opposite directions, thus causing their elongation. Cellular responses to stretching depend largely on the type and amount of load as well as on the composition of the extracellular matrix.	Myosin II, integrins, FAK, F-actin, Ifs, ZO-1, E-cadherin, Lmn A/C, Arp2/3, formin, coronin 1B, a-catenin, vinculin, collagens, elastin, fibrillin, fibulin, tenascin-C, pacsin-2, F-actin, microtubules	[26,27,28,29,30]
Compression	Contrary to tension, compressive forces applied from the outside towards the centre of cells result in cells contraction and shortening.	Collagen, vimentin, F-actin ROCK, myosin regulatory light chain, Wnt/β-catenin	[29]
Shear Stress	When two opposite forces are tangentially applied to cells surface, they generate shear stress, which cause changes in morphology and adhesion properties.	PECAM1, VEGFR, ERK1/2, PGTS2, IER3, EGR1, IGF1, IGFBP1, Integrin, TGF-β, β-catenin, MAPK, laminin-5, F-actin, PI3K	[31,32]
Hydrostatic Pressure	Hydrostatic pressure is the force exercised by the surrounding fluid to cells membranes. Due to its nondirectional nature, it is mainly non-deforming but has an important thermodynamic effect on the cytoskeleton influencing microtubule stability.	Shc1, integrins, collagen, TGF-β, F-actin	[32]
Stiffness	The term stiffness, which generally is used to describe the ability of an object to resist deformation after the application of a force, is also a measure of the rigidity of the extracellular matrix or the cells were those forces are applied.	Integrin (α2), fibronectin, collagens, α-actinin, Rho signaling cascade, talin vinculin, FAK, BMP receptor, F-Actins, vimentin Ifs, microtubules, filamin, lamin-A/C, emerin, Yap1	[28,33,34]
Elasticity	Elasticity is the property of the object to complement its original shape and size after removal of the applied force. In biology is the resistance of cells to the extracellular matrix deformation.	Collagen VI, tenascins, titin, elastin, fibrillins, integrins, F-Actins, microtubules, Myosin II	[33]
Viscoelasticity	It indicates the elastic and viscous properties by which an object contrasts the deformation.	Collagens, Elastin, ICAM-1, F-Actins	[35,36]

**Table 2:** Macromolecular complexes for mechanotransduction activity.

Extracellular Matrix	Ref.
**Solid Components Proteins** **Collagens** are the main structural glycoproteins of ECM. They interact with other ECM components and cellular integrins and exist as fibrils of 10-300 nm in diameter (e.g., types I, II, III) and reticular forms (e.g., type IV). Fibrils transmit tensile strength originated by mechanical stresses, tension, pressure and shear while type IV collagen is bound to the other ECM structural components such as laminin and fibronectin (to form the basal lamina of basement membranes). **Fibronectin** is the major dimeric fibrillar glycoprotein of ECM. It interacts with other ECM proteins, cellular membrane integrins, glycosaminoglycans, and other fibronectin molecules. **Elastin/Tropelastin** is a hydrophobic protein rich in glycine and proline. The soluble precursor tropoelastin is secreted into the extracellular space where then polymerize into insoluble elastic fibers or sheets. Elastic fibers guarantee flexibility to the structures, which can go towards withdrawal after a temporary stretch. Elastin interacts with the cellular integrins and with several ECM components (e.g., collagens, laminin, fibrillin, proteoglycans, glycosaminoglycans). **Laminins** are high-molecular-weight heterotrimeric glycoproteins formed by α, β andγ subunits that combine to form 15 different types of heterotrimers. They represent the main non-collagenous components of the basal membrane. ***Other proteins*:** vitronectin, tenascins, nidogens, fibulins, trombospondins. **Glycosaminoglycans (GAGs)** **Hyaluronic acid** is a polysaccharide consisting of alternating residues of D-glucuronic acid and N-acetylglucosamine. It is absent in proteoglycan. It confers the ability to resist compression through swelling by absorbing water. Hyaluronic acid regulates cell during embryonic development, inflammation, healing processes and tumor development. **Proteoglycans** **Chondroitin sulphate** is involved in compression of ECM. It contributes to the tensile strength of cartilage, tendons, ligaments, and affects neuroplasticity. **Heparin/Heparan sulphate** is involved in cell adhesion, migration and proliferation, developmental processes, angiogenesis, blood coagulation and tumor metastasis. It serves as a cellular receptor for a number of viruses. **Dermatan sulphate** interacts with different cell receptors and with other ECM components (e.g., collagen, tenascin, fibronectin, GAGs, and other proteoglycans). **Keratan sulphate** regulates the diameter of the fibrils in ECM and regulates interfibrillar spacing. It interacts with many proteins of the neural tissues and with collagen, glycosaminoglycans, and proteoglycans. **Syndecans** The syndecan protein family has four members that have a single transmembrane domain that act as coreceptors. These core proteins contain three to five heparan- and chondroitin-sulfate chains, which allow the interaction with different growth factors, fibronectin and antithrombin-1.	[43,71,72,73,74,75,76,77,78,79,80,81,82,83,84,85,86]
**Soluble components** **Cytokines:** TNF-a, IL-7, IL-2, CCL5, MIP-1b **Growth factors:** VEGF, FGFR1, PDGF, TGF-α, TGF-β, bFGF, IGF-1 ecc. **Matrix metalloproteinases and proteases:** adamalysins, serralysins, astacins and metzincin superfamily.	[87,88,89]
**Integrins**	
**Integrins** are the main transmembrane proteins that established cell-ECM interaction. They are heterodimers of α and β subunits. In mammals there are 18 α and 8 β subunits that associate to form 24 integrins that have affinity for different ligands. They have a large extracellular domain that links to ECM proteins and a cytoplasmic tail that bind to the cytoskeleton proteins.	[90,91]
**Focal Adhesion (FA)Proteins**	
**Vinculin** is the main protein of the FA complex. It is involved in the connexion of integrins with F-actin. Vinculin is involved in the association of cell-cell and cell-matrix junctions and is also critical in controlling the cell spreading, cytoskeletal mechanics, and lamellipodia formation. Therefore, vinculin has an essential role in controlling focal adhesions structure and function. **Paxillin** binds tubulin and targets focal adhesions through its C-terminal region, which is composed of double zinc finger LIM domains organized in four tandems. **Talin** interact with vinculin and paxillin and exists in two isoforms, talin1, ubiquitously expressed, and talin2 (striated muscle and brain). The N-terminal FERM domain have three subdomains: F1, F2, and F3. The latter contains the binding site for integrin β tails and is enough to activate integrins. **Focal adhesion kinase (FAK).** The C-terminal region contains the FAT (focal adhesion targeting) domain for the binding with proteins of the focal adhesion complex. The N-terminal domain interacts with the β1 subunit of integrins and is involved in the transduction of signals from ECM. **Other proteins:** p130Cas, zyxin, tensin, tindlins, Ena/VASP family, Arp2/3 complex.	[62,92,93,94]
**Adherens Junctions (AJs)**	
**Cadherins (N-cadherin, E-cadherin, P-cadherin, T-cadherin, V-cadherin).** Cadherins or “calcium-dependent adhesion” proteins belong to the cell adhesion molecule (CAM) family and are involved in the formation of AJs and mediate cell-to-cell contact. During development, they are essential for the proper positioning of the cells. This includes the separation of the different tissue layers and cell migration. The transmembrane domain contains five repetitions in tandem that allow the binding of Ca^2+^ ions while the extracellular domain mediates the connexion between adjacent cells. In fact, a cadherin interacts with another cadherin of the same type on the adjacent cell in an anti-parallel conformation, creating a linear adhesive “zipper” between cells. The C-terminal cytoplasmic ends, mediate the binding to catenins, which in turn interact with the actin cytoskeleton. **β-catenin** (Catenin beta-1) is a multifunctional protein involved in the transduction of Wnt signals and in the intercellular processes of adhesion by linking the cytoplasmic domain of cadherin. **α-catenin** binds cadherins and F-actin. Moreover, α-catenin recruit vinculin. **Other proteins:** l-afadin, p120, EPLIN (also known as Lima-1), ZO-1, nectins.	[95,96,97,98,99,100]
**Cytoskeleton**	
**Microtubules** are polymers of α-tubulin and β-tubulin dimers that form protofilaments, which are then associated laterally (13 protofilaments) to form a hollow tube diameter of about 25 nm. Microtubules are essential for determining cell shape and movement, intracellular transport of organelles and the formation of mitotic spindle. The dynamic activity of microtubules is under the control of microtubule-associate proteins, which increase their stability or disassembling, separation and increasing the rate of tubulin depolymerization. **F-Actin microfilaments** are polymers of G-actin monomers. F-actin fibers (diameter of about 7 nm) generate networks that regulate cellular shape and are directly involved in the generation of forces, cell migration and division. Actin filaments end at the plasma membrane, where they form a network of philopodia and lamellipodia that provide mechanical support to cells. Moreover, the activity of F-actin is strictly assisted by many actin-binding proteins. **Intermediate filaments** have a diameter of about 10 nm, have a structural role and provide mechanical strength to cells. They organize and participate to the three-dimensional structure of the cell and nucleus, and serves as anchor to organelles. Moreover, they contribute to some cell-to-cell and cell-to-matrix junctions. Intermediate filaments belong to vimentins, keratin, neurofilaments, lamins and desmin families. **Actin-Linking- Proteins** **Myosin II** is a motor protein that associate with F-actin generating both extensile and compressive forces that push and pull actin filaments by hydrolysis of ATP. **α-actinin** is a member of the spectrin superfamily. It forms an anti-parallel rod-shaped dimer by which binds both actin- domain at each end and bundles actin filaments at rod-end. **Filamins** family serve as scaffolds for more than 90 partners (e.g., channels, receptors, transcription factors) through its immunoglobulin-like domains. Filamin binds all actin isoforms (e.g., F-actin, G-actin). It forms a flexible bridge between two actin filaments generating an actin network with movable or gel-like qualities with increased elastic stiffness depending of the critical concentration of filamin. **Cofilin** protein has emerged as a key regulator of actin dynamics. In particular, it regulates the actin filament assembly/disassembly by binding to actin monomers and filaments. **Other proteins:** Arp2/3, fascin, spectrin, profilin, fimbrin (also known as is plastin 1), formins, villin	[101,102,103,104,105,106,107,108,109,110]
**Nucleoskeleton**	
**LINC complex.** **SUN1 and SUN2** are transmembrane proteins of the inner nuclear membrane with a conserved C-terminal SUN domain that localize to the perinuclear space. **Nesprins** contain the conserved KASH domain at transmembrane C-terminal tail by which bind SUN proteins. KASH–SUN bridges interact with the cytoskeleton and therefore respond to the forces generated by the cytoskeleton. **Lamins** **Lamin A/C** are intermediate filaments that ensure the nuclear architecture. They have a role in nuclear assembly, genome organization and telomere dynamics. Lamin A responds to the cytoskeletal tension and interacts with numerous proteins involved in transduction pathways. Lamin A/C expression is lower in stem cells and increases in differentiated stem cells. **Lamin B1/B2** are components of the nuclear lamina, form an outer rim and interact with chromatin. Lamin B is expressed in all cells. **Other proteins** LAP2, BAF.	[111,112,113,114,115,116]

**Table 3:** Stem cells types and properties.

Stem Cell Types	Properties	Ref.
Naïve Stem Cells	Naïve stem cells are present in the pre-implanted blastocyst cell mass and are able to generate a chimera with all types of cells present in adult tissues	[200,201,202]
Primed Stem Cells	Primed stem cells are present in the post-implantation epiblast and they cannot generate a chimera although they are capable to give rise to all types of differentiated cells.	[200,201,202]
Embryonic Stem Cells (ESCs)	These stem cells are generated from naïve embryonic stem cells in mice and primed stem cells in humans. ESCs can be differentiated into cells from all three embryonic germ layers (ectoderm, mesoderm, endoderm) and could be used as a substitute to germline stem cells for the generation of animal models.	[201,203,204,205,206,207]
Adult Stem Cells	Adult stem cells exist in pre- and post-natal organs and have self-renewal and multipotency properties. They persist within the niche of adult tissues and organs replacing cells within the tissue under physiological and pathological conditions and can be listed according to their germ layer origin Mesoderm: *Adipose Mesenchymal Stem Cells,* *Bone Marrow Mesenchymal Stem Cells, Endothelial stem cells, Hematopoietic Stem Cells, Dental Pulp Stem Cell* Endoderm: *Endothelial Stem Cells* Ectodermal: *Neural Stem Cell, Epidermal Stem Cell*	[208,209,210,211,212,213,214,215,216]
Induced Pluripotent Stem Cells (iPSCs)	iPSCs have self-renewal capacity and are pluripotent (similarly to ESCs) and they can be obtained from *in-vitro* reprogramming of somatic cells. Despite their therapeutic potential there are still obstacles for their clinical use such as teratomas formation, karyotypic abnormalities, genetic and immune rejection and immature phenotype of iPSCs-derived tissues.	[217,218,219,220]
Cancer Stem Cells	Cancer stem cells have been recognized as cells that cause tumor progression. They have self-renewal and multipotency properties and other critical features required for the metastatic development. These cells may be isolated directly from the tumor site.	[221,222]

**Table 4:** Computational Morphometric Descriptors.

Shape Descriptors	Formula	Description	References
**Parameters for Elongated Morphology**			
Aspect Ratio (AR)	$\frac{Major\;axis\;lenght}{Minor\;axis\;lenght}$	AR is defined as a ratio between the major and minor axis of the best ellipse that contains the cell. Value equal to 1 is a circle. As the ratio decreases from 1, the object becomes more elongated.	[301,302]
Eccentricity (E)	$\sqrt{1-{\left(\frac{Minor\;axis\;lenght}{Major\;axis\;lenght}\right)}^{2}}$	E is defined as a ratio between the major and minor axis of the ellipse that contains the cell. Value equal to 0 is a perfect circle. As the ratio increases from 0, the object becomes more elongated.	[300,303]
Rectangularity Factor (RF)	$\frac{Area}{\left(S*L\right)}$	RF is defined as a ratio between area and the bounding box of the cell, where S is the smaller side of the minimum bounding rectangle and L is the large side of the minimum bounding rectangle. Lower value implies a less rectangular morphology.	[303,304]
Elongation Index (EL)	$1-\frac{S}{L}$	EL is a ratio between the length and width of the object, where S is the smaller side of the minimum bounding rectangle and L is the large side of the minimum bounding rectangle. Value equal to 0 is circularly shaped. As the ratio increases from 0, the object becomes more elongated.	[305]
**Parameters for Rounded Cell Morphology**			
Cellular Shape Index (CSI)	$\frac{Area*4\pi }{perimeter^{2}}$	CSI is defined as a ratio of the object’s area to the area of a circle with the same perimeter. Value equal to 1 is a circle. As the ratio decreases from 1, the object becomes more elliptical.	[306]
Roundness (RN)	$\frac{perimeter^{2}}{Area*4\pi }$	RN is defined as the reciprocal of CSI. The minimum value is 1 for a perfect circle. An object with complicated, irregular boundaries has larger roundness.	[307]
RN/shape factor (RNF)	$\frac{4*Area}{\pi *d^{2}}$	RNF is an improvement of RN. It’s defined as the ratio between the cell area and the area of a circle with the same diameter as the cell. Value is equal to 1 in a rounded object.	[308]
**Parameters for Irregular Morphology**			
Solidity (SD)	$\frac{Cell\;Area}{Convex\;Hull\;Area}$	SD is defined as a ratio between the cell area and the convex area (smallest convex polygon that encloses the whole shape). Value equal to 1 implies a solid object. As the ratio decreases from 1, the object becomes having an irregular boundary or containing holes.	[309]
Dispersion Index (DP)	log2(π∗a∗b)	DP is defined as the binary logarithm of π∗a∗b where a is the maximum axes length and b is the minor axes length of the fitted ellipse. As the ratio increases from 0, the object become having an irregular boundary or containing holes.	[310]
Sphericity (SP)	$\frac{r}{R}$	SP is defined as a ratio between the radius of the maximum inscribed circle and the minimum circumscribed circle. The maximum value equal to 1 implies a spherical shape.	[311]
Spreading Index (SI)	$\frac{\pi *Convex\;Hull\;Perimeter^{2}}{4*Convex\;Hull\;Area}$	SI is defined as a ratio between the convex perimeter and the convex area. Larger values imply more elongated structures.	[312]

## Abbreviations

ADF	Actin depolymerizing factor
Arp2/3	Actin Related Proteins 2/3
BAF	Barrier-to-autointegration factor
bFGF	basic Fibroblast growth factor
BMP	Bone Morphogenetic Protein
*CCL5*	C-C motif chemokine 5
EGR1	Early Growth Response 1
EPLIN	Epithelial protein lost in neoplasm
ERK1/2	Extracellular signal-regulated kinases
FAK	Focal Adhesion Kinase
FERM	4.1 protein, Ezrin, Radixin, Moesin
FGFR1	Fibroblast growth factor receptor 1
ICAM-1	Intracellular Adhesion Molecule 1
IER3	Immediate Early Response 3
IFs	Intermediate Filaments
IGF1	Insulin-like Growth Factor 1
IGFBP1	Insulin-like Growth Factor Binding Protein
IL-2	Interleukin-2
IL-7	Interleukin-7
KASH	Klarsicht, ANC-1, Syne Homology
LAP2	Lamina-associated polypeptide 2
LIM	Lin-11, Isl-1, Mec-3
Lmn A	Lamin A
Lmn B	Lamin B
LmnC	Lamin C
MAPK	Mitogen activated protein kinase
MIP-1b	Macrophage inflammatory protein-1b
NKX-2.5	Homebox protein Nkx-2.5
p120	p120-catenin
PDGF	Platlet derived growth factor
PECAM1	Platlet And Endothelial Cell Adhesion Molecule 1
PGTS2	Prostaglandin-endoperoxide synthase 2
PI3K	Phosphoinositide 3-kinase
ROCK	Rho-associated protein kinase
*SHC1*	SHC-transforming protein 1
SUN	Sad1p, UNC-84
SUN1	SUN domain-containing protein 1
SUN2	SUN domain-containing protein 2
TAZ	Trascriptional coactivator with PDZ-binding motif
TGF-α	Transforming Growth Factor α
TGF-β	Transforming Growth Factor β
TNF-a	Tumor necrosis factor-a
VE-Chaderin	Vascular Endothelial cadherin
VEGF	Vascular endothelial growth factor
VEGFR	Vascular endothelial growth factor receptor
YAP	Yes-Associated Protein
ZO-1	Zona Occlusens-1
